# Adenovirus Recruits Dynein by an Evolutionary Novel Mechanism Involving Direct Binding to pH-Primed Hexon

**DOI:** 10.3390/v3081417

**Published:** 2011-08-12

**Authors:** Julian Scherer, Richard B Vallee

**Affiliations:** Department of Pathology and Cell Biology, Columbia University, Physicians and Surgeons Building, Room 15-409, 630 West 168th Street, New York, NY 10032, USA; E-Mail: js3203@columbia.edu

**Keywords:** adenovirus, molecular motors, cytoplasmic dynein, intracellular motility

## Abstract

Following receptor-mediated uptake into endocytic vesicles and escape from the endosome, adenovirus is transported by cytoplasmic dynein along microtubules to the perinuclear region of the cell. How motor proteins are recruited to viruses for their own use has begun to be investigated only recently. We review here the evidence for a role for dynein and other motor proteins in adenovirus infectivity. We also discuss the implications of recent studies on the mechanism of dynein recruitment to adenovirus for understanding the relationship between pathogenic and physiological cargo recruitment and for the evolutionary origins of dynein-mediated adenovirus transport.

## 1. Introduction—Role of Microtubule Motors in Adenovirus Transport

**Figure 1.**
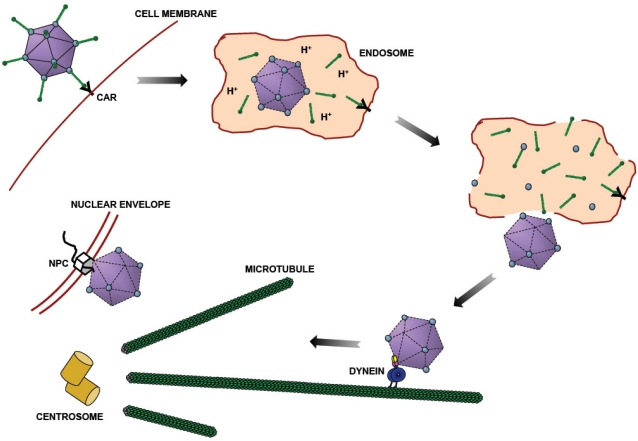
Adenovirus Entry Pathway. After binding to the plasma membrane through the Coxsackie and Adenovirus (CAR) receptor, adenovirus is taken up by endocytosis [8,9]. Some capsid proteins are lost in the acidic endosomal lumen [10–12]. Following endosomalysis, adenovirus moves bidirectionally along microtubules (MTs) [13], using dynein for transport towards MT minus ends, which are typically focused at the centrosome and the vicinity of the nucleus. Finally, adenovirus binds to the nuclear pore complex (NPC) [14] through which it injects its genome for viral reproduction.

Adenoviruses consist of nonenveloped, double-stranded DNA-containing capsids that commonly cause mild and self-limited infections in healthy individuals, but can be fatal for immunocompromised patients. At least 55 serotypes, which are divided into seven subgroups (A–G), have been identified since adenoviruses were first isolated from adenoid tissue in 1953 [1]. The individual serotypes share a similar structure, which has been solved at near atomic resolution for the prototypical subgroup C adenovirus 5 (Ad5) by cryo-electron microscopy and for an Ad5/Ad35 chimera by x-ray crystallography [2,3]. The protein shell of the virus has a diameter of 90–100 nm and consists of three major and four minor capsid proteins [4]. The trimeric fiber protein protrudes from the capsid and can interact with the Coxsackie and Adenovirus Receptor (CAR) (Figure 1). Fiber is attached to the capsid through the pentameric penton base, which is situated at each of the vertices of the icosahedral capsid. The other major capsid protein, hexon, is the most abundant subunit with 240 hexon trimers in each virus, 12 of which comprise each facet of the icosahedral virion.

The extreme resistance of adenovirus to pH, temperature, and ionic strength [5] is likely due to the tight quarternary structure of hexon [6] and the contributions of additional minor capsid proteins, such as protein IIIa, VI, VIII and IX (reviewed in Vellinga *et al.* [7]) that act as “cement proteins”, bridging gaps between the major components. Inside the capsid are the 36 kb virus genome, the viral protease, and the DNA-associated proteins V, VII, μ, and the terminal protein.

For most adenovirus serotypes, uptake into the cell is initiated by binding to CAR via the knob domain at the tip of the fiber protein (Figure 1) and then strengthened by further interaction between the penton base RGD motif with cell surface-associated α5 integrins. These binding events lead to endocytosis of the capsid into clathrin-coated pits [8,9] and activation of PKA and p38/MAPK [15]. Inside the endosome, acidification to pH 4.6–6.0 [16] has been found to induce a number of structural changes in the virion, consistent with low pH-dependent structural effects *in vitro* [17]. Fiber proteins are shed from the capsid [10], and protein VI leaves the inside of the capsid and is presumably released into the endosomal lumen [11]. The amino-terminal end of protein VI has an amphipathic helix that then partially ruptures the endosomal membrane by inducing positive curvature in the inner leaflet of the lipid bilayer [18], thus allowing the capsid to escape into the cytoplasm. pH-dependent changes in the conformation of the remaining capsid proteins, including hexon, have also been described [17].

By about 15 minutes post-infection (p.i.), adenovirus subgroups A, C, D, E, and F have escaped the early endosome [19], whereas subgroup B adenoviruses remain in the endosomal/lysosomal pathway for up to eight hours [20,21]. Within 30–45 min after endosomal escape, subgroup C adenovirus reaches the nuclear envelope or the vicinity of the centrosome, depending on cell type. That this redistribution might involve microtubules (MTs) and MT-based transport was initially suggested by EM images of adenovirus-infected HeLa cells, which revealed partial coincidence of capsids with MTs [22,23]. More direct evidence came from live cell analysis of cytoplasmic viruses, which revealed capsid transport along linear trajectories consistent with the organization of MTs [13,24,25]. Furthermore, the use of nocodazole to depolymerize MTs in infected cells strongly inhibited adenovirus redistribution and directed transport [13,24]. Infectivity was also found to be markedly reduced, indicating an essential role for microtubules in the adenovirus infectious cycle [26].

Live cell analysis of virions after endosomal escape has indicated capsid transport along MTs in both the MT minus- and plus-end directions, presumably driven by cytoplasmic dynein and kinesin motors, respectively. While little is known about the function of kinesins in adenovirus transport (discussed below), interference with cytoplasmic dynein by RNAi, microinjection of function blocking antibodies, and expression of dynein inhibitors (dynamitin, and truncation mutants of the dynactin subunit p150^Glued^ and the dynein heavy chain) strongly inhibited adenovirus redistribution to the cell center based on fixed and live cell analysis [13,24,25]. Adenovirus interacts with the nuclear pore protein CAN/Nup214 [14], but can accumulate in the cell center even in enucleated lung endothelial cells [27], illustrating the importance of MT minus end transport alone in adenovirus redistribution.

Because endosomes are subject to microtubule-mediated transport, adenovirus motility could to some extent reflect the behavior of the enclosing vesicular structures. Several lines of evidence, however, indicate an association of capsids with dynein after endosomal escape. Adenovirus tagged with a pH-sensitive dye revealed the particles to be at neutral pH by 30–40 min following infection [24], and almost no particles colocalized with early endosomal markers at this time [25], but most viruses showed positive immunoreactivity using antibodies to several dynein subunits and regulatory factors. These data, therefore, suggest that endosomal escape occur before adenovirus particles are typically imaged for directed transport.

## 2. Identification of Viral Capsid Proteins Involved in Cytoplasmic Dynein-Mediated Motility

The latter evidence indicates that following endosomal escape, adenovirus binds dynein as a naked capsid. The number of proteins potentially exposed on the capsid surface is limited (hexon, penton base, fiber, proteins IIIa, VI, VIII, IX), and candidates for a dynein interaction are further reduced by the stepwise dismantling of the virus and shedding of capsid components that occurs during early entry steps. Fiber is lost close to the cell surface. Penton base molecules are substantially removed during passage through the endosome [10,12], though this subunit can still be detected in association with nuclear envelope-associated virus particles [25]. The smaller capsid proteins IIIa, VI, and VIII are internal [2,3] and are mostly lost from the virus core within the first 20 min after infection [11,12,28]. Nevertheless, a mutation in the protein VI ubiquitinylation sequence has been found to reduce redistribution of incoming virus to the nucleus [28]. However, whether protein VI participates directly in dynein-mediated virus transport remains uncertain, because the protein is thought to remain associated with only a minority of cytoplasmic virus particles [11,12,28]. Protein IX is the only minor capsid protein accessible on the outer surface of the virion, and it also remains with the virus genome until its delivery into the nucleus [29]. An adenovirus mutant lacking protein IX, however, shows the same intracellular motility characteristics as the wild-type virus [30], arguing against a role in motor recruitment. Hexon is the most abundant capsid protein, and remains associated with the viral DNA until virus attachment to the nuclear pore complex [12,14,31,32].

To address more directly the mechanisms responsible for mediating the dynein-adenovirus interaction, we conducted a series of biochemical tests using whole purified brain cytoplasmic dynein, virus capsid, and individual capsid proteins [25]. We found that, among the reasonable candidate proteins, only hexon showed evidence of an interaction with cytoplasmic dynein. Penton base, proteins V, VII, and X were all negative in these tests. The behavior of hexon, itself, proved to be highly dependent on solvent conditions. However, once this variable was identified, robust interaction with dynein was observed, as discussed below. In addition, infection of HeLa cells heterologously expressing hexon displaced dynein from incoming adenovirus *in vivo* [25].

## 3. pH-Dependent Biochemical Characteristics of the Adenovirus Capsid and Hexon

Adenovirus purified by conventional CsCl banding has been reported to show some affinity for microtubules, which could be diminished in the presence of ATP or in the absence of MAPs, consistent with involvement of a motor protein [33]. However, we observed no detectable interaction of purified virus with cytoplasmic dynein at neutral pH [25]. To test whether the virus might be primed for cytoplasmic transport by its passage through the endosomal pathway, we exposed purified Ad5 to acidic pH conditions, and then returned the virus to neutrality. We observed a clear enhancement in dynein binding after exposure to pH levels in the 4.4–5.4 range. These values are based on conditions reported for intracellular adenovirus-containing acidic vesicles [16] and are somewhat lower than the range reported for early endosomes of uninfected cells (pH5.0 to pH6.2) [34–36].

**Figure 2.**
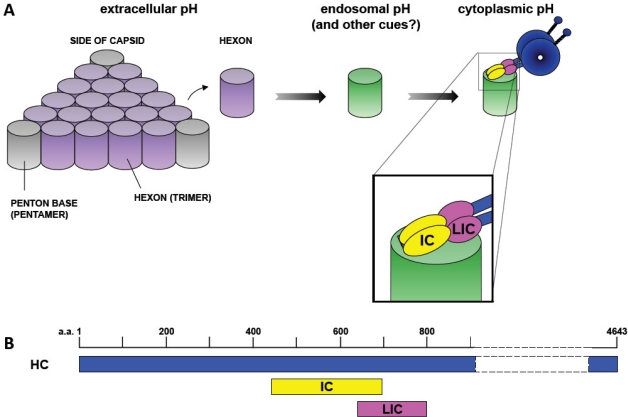
Mechanism of Dynein Recruitment to Adenovirus. (**A**) At extracellular pH values (indicated by purple shading), adenovirus interacts weakly if at all with cytoplasmic dynein. Short-term transient exposure of virus or purified hexon trimer to the low pH values (green shading) characteristic of the endosomal lumen results in stronger dynein binding upon return to neutrality. Hexon binds directly and specifically to the dynein intermediate (IC) and light intermediate (LIC) chains [25]. (**B**) Representation of the binding sites of the dynein intermediate and light intermediate chains on the dynein heavy chain (HC) [41]. ICs and LICs associate with contiguous sites within the tail domain of the dynein complex and could, potentially, provide a continuous binding interface for hexon. Numbers indicate amino acid residues.

We also tested for dynein binding by hexon alone, typically immunoisolated from lysates of Ad5-infected cells, but also purified chromatographically [37]. Hexon mimicked the behavior of the intact virus, showing a clear ability to pull down purified dynein, but only after low pH priming (Figure 2). These results strongly support a mechanism for dynein recruitment to adenovirus involving pH-dependent priming of hexon in the endosome. The correlation in the behavior of hexon with that of intact capsids is an additional piece of evidence supporting a predominant role for this subunit. The effects of reduced pH on dynein recruitment are in addition to other consequences of low pH exposure on infectivity such as rearrangement of capsid composition and possibly endosomal membrane disruption [11,17,38]. Thus, our data are consistent with physiological evidence for the importance of adenovirus exposure to endosomal pH for efficient infection (though see Smith *et al.* [39]). We also point out that exposure of hexon to low pH may be a useful mechanism to ensure differential transport of incoming virus capsids *vs.* newly expressed hexon trimers later in infection. At this stage hexon trimers redistribute from the cytoplasm to the nucleus using the nuclear localization signal of protein VI for nuclear targeting. No role for direct MT-based hexon transport in this process has been reported [40].

The nature of the low pH effect on hexon structure or conformation is uncertain. Hexon has been reported to exhibit an increase in hydrophobicity at pH values below pH 5.5 as judged by solvent partitioning experiments using Triton X-114 [17] and an increase in susceptibility to proteolysis [42]. These reports support a pH-induced conformational change, but the molecular details underlying this mechanism remain to be explored more fully.

## 4. Identification of Hexon-Interacting Cytoplasmic Dynein Subunit

Cytoplasmic dynein is a complex of two copies of a heavy chain (HC); intermediate chains (IC1 and IC2); light intermediate chains (LIC1 and LIC2); and three classes of light chains (LC8, TcTex, and LC7/Roadbock) [43,44]. The HC consists of a C-terminal motor domain (∼380 kDa) and an N-terminal tail domain, which is responsible for self-association and cargo binding, which, in turn, involves the ICs, LICs, and LCs. The ICs form a dimeric subcomplex, which is able to sequester the LCs and appears generally to block their interaction with most if not all other known binding partners [45,46]. This arrangement argues against a role for the LCs in directly linking cytoplasmic dynein to other proteins, but, instead, a role in modulating the stability or other conformational properties of the ICs. The extent to which the ICs serve directly in dynein cargo binding remains incompletely explored [47]. However, the ICs and LCs interact with two important dynein regulatory complexes, dynactin [48,49] and LIS1-NudE/NudEL [50], each of which, in turn, are involved in physiological cargo binding as well as dynein motor regulation.

The LICs are the least well studied of the cytoplasmic dynein subunits. They have been implicated in specific aspects of dynein cargo binding, interacting with pericentrin, Par3, and Rab11-FIB3 [51–53]. However, they have general roles in lysosome/late endosome motility and mitosis [54,55], though the mechanisms underlying these functions are incompletely understood.

To test which of these dynein components are involved in recruitment to adenovirus we used lysates from cultured mammalian cells overexpressing individual dynein polypeptides [25]. These studies reveal that the products of both dynein IC1 and IC2 genes and LIC1 are able to bind to acid-primed immunopurified hexon (Figure 2). LIC2 and the dynein light chains TcTex-1, RP3 and LC8 were negative in these assays [25].

## 5. Involvement of Cytoplasmic Dynein Regulators in Adenovirus Transport

Cytoplasmic dynein is regulated by a diversity of factors. The two best known and most extensively studied are the dynactin complex and a complex of NudE or NudEL with the product of the smooth brain gene, LIS1. Dynactin, NudE and NudEL have each been implicated in dynein recruitment to physiological forms of cargo, such as Golgi elements, components of the endosomal pathway, the G2 nuclear envelope, and mitotic kinetochores [50,56–59]. In addition, these factors regulate dynein motor behavior. Dynactin has been found to stimulate dynein processivity *in vitro* [60,61]. NudE and LIS1 together modify dynein to participate in high force functions, such as nuclear migration [62].

Immunocytochemical analysis revealed that both dynactin and NudE/NudEL colocalize along with cytoplasmic dynein on a high percentage of post-endosomal adenovirus particles (∼80%) in infected cells, though LIS1 colocalization was quite low. To test whether the NudE, NudEL, and dynactin interactions with virus were direct, adenovirus was used in pull downs from infected cell lysates [25]. Cytoplasmic dynein clearly associated with the virus particles in these assays, but dynactin and NudE/NudEL were undetectable. Furthermore, neither purified dynactin [63] nor recombinant NudE [62] showed an interaction with acid-primed hexon [64]. Together with our evidence for an interaction between purified dynein and virus, these results suggest that dynactin and NudE or NudEL are not involved in dynein recruitment to virus, but may, nonetheless, play regulatory roles in virus transport.

Interference with dynactin function has repeatedly been found to inhibit virus redistribution toward the cell center [13,25]. Whether this is due to a decrease in virus run-length along MTs or to a loss of dyneins from the capsid surface has been uncertain, though expression of the CC1 dynactin fragment has been found to reduce virus travel distance toward MT plus ends [65]. However, inhibition of NudE/NudEL and LIS1 by injection of function-blocking antibodies or expression of dominant negative fragments showed no obvious effect on the overall redistribution of virus to the cell center [25]. These results support an important role for dynactin in virus transport, but reveal no apparent role for NudE, NudEL, and LIS1. Why NudE and NudEL are associated with virus particles *in situ* remains to be resolved, but it is possible that they represent passive passengers during virus transport. The presence of NudE alone is inhibitory to dynein motility in *in vitro* single molecule and biochemical assays [62], and an association with adenovirus could imply a minimal or even inhibitory role in virus transport. Hence, blocking NudE/NudEL function might, potentially, result in faster capsid transport towards the nucleus. Recruitment of LIS1 to the virus particle might be as needed, e.g., during transport through cell constrictions or crowded cytoplasmic regions.

ZW10, an additional dynein recruitment factor, which interacts with dynein directly [66] or through dynactin [67,68], showed minimal colocalization with incoming adenovirus. Furthermore, ZW10 RNAi had no effect on virus progression towards the nucleus [25].

## 6. Evolutionary Origin of Dynein Recruitment by Viruses

**Figure 3.**
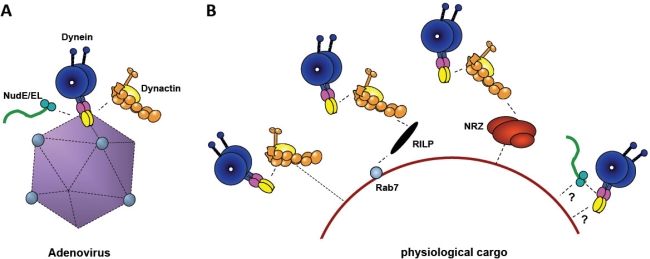
Comparison of Adenovirus-mediated Dynein Recruitment with Physiological Mechanisms. (**A**) Cytoplasmic dynein is shown binding to adenovirus directly, and dynactin (yellow) and NudE/NudEL (green), indirectly. (**B**) Physiological cargoes (e.g., lysosomes, as shown) feature a variety of different dynein recruitment mechanisms, several involving dynactin. Both dynactin and NudE/NudEL are required for motility, and may, potentially, contribute either to dynein recruitment or to mechanochemical regulation, or both.

Several features of the adenovirus-dynein interaction suggest an evolutionary origin independent of existing interactions between the motor protein and physiological forms of cargo (Figure 3). The latter is proving to be very complicated, and the complete mechanisms have not been worked out for any individual physiological form of cargo. It is known, however, that dynein is commonly attached to cargo through recruitments factors, such as dynactin, NudE, NudEL, and ZW10, which may, in turn, be recruited by additional factors (Figure 3B). In contrast, at least some reported LIC interactions (Par3, pericentrin, Rab4, and the Rab11 effector FIP3 [51–53]), or IC interactions (beta-catenin [47]) may be a manifestation of direct cargo-recruitment functions.

Our own recent evidence indicated that adenovirus interacts with dynein directly and through IC and the LIC1 subunits (Figure 3A). Both the directness of the interaction and the involvement of two different dynein subunits in dynein recruitment to a single form of cargo is atypical. It is of interest that the ICs and LICs are arrayed in tandem within the tail portion of the dynein HC [41] (Figure 2). This arrangement raises the possibility that the two dynein subunits might provide a contiguous binding surface for adenovirus. African swine fever and herpesviruses also seem to interact directly with cytoplasmic dynein [69,70]. In the case of African swine fever virus, it has been reported that the interaction is mediated through the dynein light chain LC8 and the viral p54 protein and can be selectively inhibited by a small LC8-derived peptide [71]. However, p54 and the dynein ICs appear to bind to a common groove within the LC8 dimer [45,71]. Herpes virus proteins of the inner tegument are the most likely candidates to recruit cytoplasmic dynein. Interestingly, the same pool of proteins has also been shown to bind directly and independently to dynactin and kinesins [70]. The functional consequences of earlier reports indicating an interaction between the herpesvirus protein VP26 (UL35) and the dynein light chains RP3 and TcTex-1 or UL34 with the dynein intermediate chain remain to be fully resolved [72–74].

Preliminary evidence suggests that the binding site for adenovirus hexon within the dynein IC is non-overlapping with that for dynactin and NudE/NudEL [75]. This arrangement should allow dynein binding to adenovirus while preserving dynein interactions with its regulators. Thus, despite the ability of adenovirus to recruit dynein directly, dynactin is still important for transport. A role for dynactin has also been reported for Herpes simplex virus and African swine fever virus [69,70,76], suggesting that dynactin may be generally required for virus transport. As noted above, there is at present no known role for NudE and NudEL in adenovirus transport, despite their clear colocalization with capsids after endosomal escape.

We also note that, although expression of hexon in cultured mammalian cells interferes with dynein recruitment to adenovirus capsids and their transport to the nucleus, hexon had no effect on Golgi morphology [25]. These results support distinct, noncompeting interactions of the dynein LIC1 and IC subunits with hexon *vs.* physiological forms of cargo, and argue that development of reagents to block virus transport specifically is feasible.

## 7. Bidirectional Intracellular Adenovirus Motility

Post-endosomal adenovirus exhibits bidirectional linear movements along microtubules [13,15,24,25]. These observations suggest that capsids are transported not only by cytoplasmic dynein but also by at least one form of kinesin. However, microinjection of established function-blocking anti-kinesin-1 monoclonal antibodies had no effect on virus redistribution [24]. Thus, the molecular mechanism responsible for plus end directed adenovirus transport remains unresolved. However, alpha- and gammaherpes viruses have been found to interact with kinesin-1 and -2 [70,77,78]. Vaccinia virus has been reported to recruit kinesin-1 heavy chain directly via an interaction with the F12 subunit, which structurally mimics the kinesin light chain [79]. Furthermore, parvovirus, influenza virus, human foamy virus, and human immunodeficiency virus also show directed translocation along MTs [80–83].

The physiological significance of bidirectional virus transport is not fully understood. Plus end movement might represent part of the host response to prevent virus from reaching the nucleus [84]. This model seems reasonable, as the virus would be expected to reach the nucleus much more efficiently using dynein alone. It has been suggested, however, that virus reaching the centrosomal region of the infected cell may still require kinesin for microtubule plus end directed transport to the nuclear envelope [85,86]. Analysis of adenovirus motility using high-resolution particle tracking revealed a near-zero net flux in the direction of virus movement from the periphery to the center of infected cells [13,25,30]. This result raises the possibility that viruses may reach the cell center via an assisted random walk mechanism. In this view motor proteins may allow virus particles to rapidly and more fully explore the cytoplasm until they encounter a nuclear pore complex, to which they bind with high affinity.

## 8. Conclusions

Adenoviruses rely on minus end directed transport along MTs by cytoplasmic dynein for efficient infection of their host cells, as seems to be true for at least some and likely many other pathogens. Adenovirus seems not to have modified existing physiological dynein recruitment mechanisms for its own use. Rather, it developed an unusual, if not unique, mechanism for hijacking dynein and using it to hitchhike to the nucleus. This insight may allow for development of specific inhibitors of viral infections with minimal effect on normal cellular behavior. A molecular understanding of motor recruitment also provides an inventory of factors, which must be preserved for successful use of adenovirus as a vector in gene therapy and vaccination trials. Adenovirus vectors are increasingly being used as therapeutic agents because of their large capacity for extra-genomic DNA as well as their ability to infect dividing and non-dividing cells. Preservation and improvements in the efficiency of adenovirus may be of great value in these efforts.
